# Interactions between Host Immunity and Skin-Colonizing Staphylococci: No Two Siblings Are Alike

**DOI:** 10.3390/ijms20030718

**Published:** 2019-02-07

**Authors:** Young Joon Park, Chae Won Kim, Heung Kyu Lee

**Affiliations:** 1 Graduate School of Medical Science and Engineering, Korea Advanced Institute of Science and Technology (KAIST), Daejeon 34141, Korea; youngjoon.park@kaist.ac.kr; 2 Biomedical Science and Engineering Interdisciplinary Program, KAIST, Daejeon 34141, Korea; chaewon.kim@kaist.ac.kr; 3 KAIST Institute for Health Science and Technology, KAIST, Daejeon 34141, Korea

**Keywords:** cutaneous immunity, microbiome, *Staphylococcus* spp., T cells, *Staphylococcus aureus*, *Staphylococcus epidermis*, commensals, atopic dermatitis

## Abstract

As the outermost layer of the body, the skin harbors innumerable and varied microorganisms. These microorganisms interact with the host, and these interactions contribute to host immunity. One of the most abundant genera of skin commensals is *Staphylococcus*. Bacteria belonging to this genus are some of the most influential commensals that reside on the skin. For example, colonization by *Staphylococcus aureus*, a well-known pathogen, increases inflammatory responses within the skin. Conversely, colonization by *Staphylococcus epidermis*, a coagulase-negative staphylococcal species that are prevalent throughout the skin, can be innocuous or beneficial. Thus, manipulating the abundance of these two bacterial species likely alters the skin microbiome and modulates the cutaneous immune response, with potential implications for various inflammation-associated skin diseases. Importantly, before researchers can begin manipulating the skin microbiome to prevent and treat disease, they must first fully understand how these two species can modulate the cutaneous immune response. In this review, we discuss the nature of the interactions between these two bacterial species and immune cells within the skin, discussing their immunogenicity within the context of skin disorders.

## 1. Introduction

The skin is the largest organ of the body and is inhabited by millions of commensals, including bacteria, fungi, and viruses. Despite their abundance, whether these microorganisms benefit the host is unclear. Recent technological advances such as genome sequencing have expanded our understanding of the composition and function of the skin microbiome. We now know that the microorganisms which reside in our body fall on a spectrum from ‘good’ to ‘troublesome’ residents. Specifically, beneficial microflora (the ‘good’ residents) usually cause no secondary infections, but cannot be eliminated as causes of illness [1]. Alternatively, troublesome residents are generally considered pathogenic but can sometimes aid the host by preventing colonization by other microorganisms.

In the skin, one of the most studied troublesome residents is *Staphylococcus aureus*. These bacteria have long been notorious pathogens. *S. aureus* belong to the genus *Staphylococcus*, which is comprised of fifteen unique species that are differentiated based on molecular data [2]. Whether *S. aureus* should be considered a commensal is controversial; however, approximately 30% of the human population carries asymptomatic *S. aureus* in their nose [3]. Healthy control skin also carries *S. aureus*, indicating that the presence of *S. aureus* is not always directly associated with disease [4]. Comprehensive research evaluating different species of *Staphylococcus* has shown that species other than *S. aureus* can also interact with hosts. One such species that has been the subject of recent research is *Staphylococcus epidermidis*. As members of the same genus, *S. aureus* and *S. epidermidis* share key phenotypic properties [2]. However, *S. epidermidis* belongs to a group known as coagulase-negative staphylococci (CoNS). As the name suggests, *S. epidermidis* do not possess coagulase, a blood-clotting enzyme, which distinguishes these bacteria from *S. aureus*. In addition, unlike *S. aureus*, *S. epidermidis* are generally good residents. They can also interact with the environment, and these interactions often benefit host immunity. It is interesting that although these two bacteria belong to the same genus, their effects are antagonistic. Several proteins expressed by these microorganisms, and their immunogenicity, have been uncovered. However, more research is needed regarding the biochemical characteristics and functions of these microorganisms. In this review, we provide an overview of what is currently known regarding the effects of skin colonization by *S. epidermidis* and *S. aureus* on protein production and host cutaneous immunity.

## 2. *S. aureus*

Before addressing the interactions between *S. aureus* and local immunity, we must first note that the interactions as a member of the skin microbiome differ from deep or systemic staphylococcal infections. Indeed, interactions as a resident microorganism are confined to only a minor breach of the cutaneous barrier. Soft tissue staphylococcal infections and biofilm infections on implanted medical devices are beyond the scope of this review.

**Figure 1:**
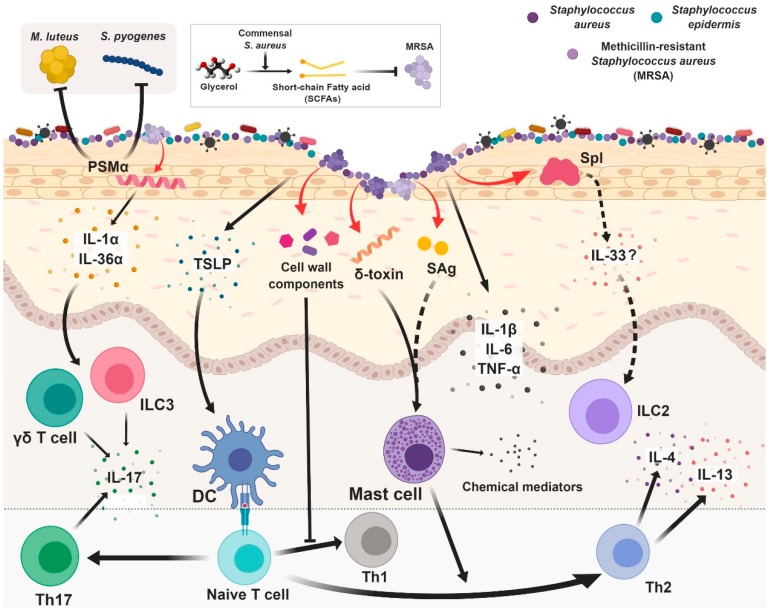
Interaction between *Staphylococcus aureus* and skin immune system. The skin immune system comprises a complex network of cells. Because keratinocytes serve as sensors, phenol-soluble modulin α (PSMα) are capable of inducing epidermal keratinocytes to produce IL-1 and IL-36, even without epidermal disruption. T cells and innate lymphoid cells (ILC3) containing the IL-36 receptor recognize IL-13 and secrete IL-17, leading to inflammation. Another peptide belonging to the PSM family, δ-toxin, promotes Th2 responses by activating mast cells. It is likely that staphylococcal superantigens (SAgs) are also capable of mast cell activation. Thymic stromal lymphopoietin (TSLP) can drive Th2 skewing via dendritic cells (DCs). Cell wall components from *S. aureus* inhibit Th1 responses. *S. aureus* also possesses probiotic properties. *S. aureus* shows antimicrobial activity against other bacteria, such as *M. luteus* and *S. pyogenes*. Commensal *S. aureus* inhibits methicillin-resistant *S. aureus* (MRSA) using short-chain fatty acids (SCFAs) fermented from glycerol. Red arrows indicate bacteria-originated materials. T-bar indicates inhibitory activity.

As a resident microorganism, *S. aureus* colonization is associated with various skin diseases, including atopic dermatitis (AD). Even before DNA sequencing technology emerged, *S. aureus* was known to be more frequent in AD patients [5]. Real-Time PCR (RT-PCR) studies confirmed the increased prevalence of *S. aureus* in the skin lesions of AD patients [6]. AD is generally considered an inflammatory skin disorder with Th2 skewing. Thus, the relationship between *S. aureus*, inflammation, and Th2 responses has been brought to the attention of those who study *S. aureus* and AD (Figure 1).

Importantly, *S. aureus* colonization noticeably affects cutaneous immunity. In barrier-disrupted murine skin, *S. aureus* colonization increases the expression of the pro-inflammatory cytokines IL-1β, IL-6, and TNF-α [7]. Upon entering the dermis, *S. aureus* increases mRNA expression of IL-4, IL-13, CXCL2, TSLP, IL-17, and IL-22, but decreases expression of cathelicidin, an antimicrobial peptide [8]. This penetration is enhanced by protease-induced *S. aureus*. *S. aureus* can also modulate cutaneous immunity through production of staphylococcal serine protease-like proteins (Spls). Spls are a group of secreted bacterial proteases that are currently known for their allergenic properties in mice and humans. When stimulated with Spls, human peripheral blood T cells produce significant amounts of Th2, but not Th1/Th17, cytokines [9]. One such Spl, SPID, induces type 2 airway responses in an IL-33-dependent manner [10]. Thus, Spls may underlie the association between *S. aureus* colonization and Th2 responses in AD.

Unlike *S. epidermidis*, *S. aureus* secrete toxins. One such toxin, δ-toxin, is known to promote Th2 responses [11]. Skin colonization with *S. aureus*, but not with a mutant deficient in δ-toxin, promoted immunoglobulin-E (IgE) and interleukin-4 production, as well as inflammatory skin disease. δ-toxin induces degranulation of dermal mast cells and increases IgE, which further enhances mast cell degranulation. This cycle may also contribute to Th2 skewing.

Furthermore, *S. aureus* membrane components and diacylated lipopeptide induce keratinocyte production of thymic stromal lymphopoietin (TSLP), which is an important initiator of AD [12]. *S. aureus* cell wall components also downregulate IP-10, trigger activation of MAPK, p38, and ERK, and inhibit STAT1 signaling in monocytes, all of which may contribute to the abrogation of Th1 cell-recruiting chemokines [3].

*S. aureus* also express superantigens (SAgs), including toxic shock syndrome toxin-1 and staphylococcal enterotoxin, that cross-bridge major histocompatibility class II molecules on antigen presenting cells and the T cell receptor on T cells. This binding strongly promotes T cell proliferation the release of Th1 cytokines. When applied to human skin, staphylococcal enterotoxin B (SEB) promotes cutaneous T cell accumulation and an AD-like response [13]. Whether this is a Th1 or Th2 response is unclear, but SAgs can interact directly with mast cells and induce T cells to secrete IL-31 [14]. IL-31 inhibits keratinocyte differentiation, downregulates filaggrin expression, and causes an itching sensation, all of which are implicated in AD development [14,15].

Finally, recent reports indicate that *S. aureus* colonization may also contribute to psoriasis and new-onset pediatric AD, both of which are associated with a Th17 rather than a Th2 response [16]. In these studies, epicutaneous *S. aureus* exposure for one week induces skin inflammation. *S. aureus* produces phenol-soluble modulin α (PSMα), which triggers IL-36R/MyD88 signaling. Consequently, IL-36 from epidermal keratinocytes stimulates T cells, which generate IL-17 [17,18]. As human AD skin has increased IL-36α/γ transcripts and an increased number of Th17 cells [19,20], excess PSMs from *S. aureus* may also be responsible for Th17-polarized skin inflammation.

*S. aureus* PSMs, nonetheless, may be of advantage to the host. PSM derivatives from PSMα1 and α2 display antimicrobial activity against *M. luteus* and *S. pyogenes*, a component of the normal flora and a highly pathogenic bacterium, respectively. Contrary to expected results, lantibiotics (lanthionine-containing peptide antibiotics produced by bacteria) did not show antimicrobial activity in the study [21].

Recently, a commensal *S. aureus* strain isolated from human perinasal skin demonstrated the ability of fermenting glycerol to release short-chain fatty acids (SCFAs), which are known for their bactericidal activity. USA300, a representative methicillin-resistant strain of *S. aureus*, does not possess such ability. When commensal *S. aureus* and methicillin-resistant *S. aureus* (MRSA) with/without 2% glycerol were applied onto the skin of wounded mice, the number of MRSA was significantly less in wounds inoculated with bacteria and glycerol than in those inoculated without glycerol. Furthermore, immunization of mice with recombinant α-hemolysin from commensal *S. aureus* yielded high antibody titers (>1:512,000), which provided protection against MRSA skin infection. These data suggest that commensal *S. aureus* bacteria may provide benefits to the host by maintaining host probiotic activity, whereas this characteristic has not been evolutionarily conserved in MRSA [22].

## 3. *S. epidermidis*

**Figure 2:**
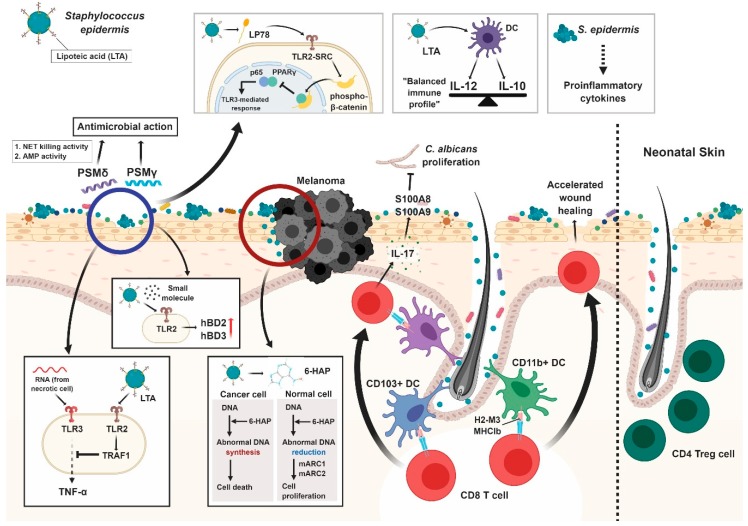
Interaction between *Staphylococcus epidermis* and skin immune system. When skin is injured, lipoteichoic acid (LTA) from the skin commensal *S. epidermidis* inhibits inflammatory cytokine production via inhibition of TLR3 signaling by TRAF1 activated through TLR2. *S. epidermidis* also produces small molecules that increase anti-microbacterial peptides through TLR2, which in turn inhibits pathogen growth. Lipopeptide 78 activates TLR2-SRC signaling in keratinocytes to induce β-catenin phosphorylation. Dissociation between p65 and PPARγ due to phospho-β-catenin binding reduces TLR3-mediated inflammation. Phenol-soluble modulin γ (PSMγ) and PSMδ from *S. epidermidis* also have anti-microbacterial activity. 6-HAP, a nucleobase analogue capable of selective tumor growth suppression was recently found to be produced by *S. epidermidis*. Cancer cells did not have sufficient mARC1 and mARC2 to reduce 6-HAP, leading to cell death. CD103+ dendritic cells (DCs) and CD11b+ DCs cooperate in tuning *S. epidermidis*-specific CD8+ T cells to protect from pathogen infection via upregulation of alarmins (S100A8 and S100A9). The commensal-specific CD8+ T cell response is induced by non-classical MHC class I molecules (H2-M3 MHCIb), and is immunoregulatory and tissue-repairing in nature. Dendritic cells (DCs) exposed to LTA preparations from *S. epidermidis* (epi-LTA) show an IL-10-balanced immune profile, with the ratio of IL-12p70 to IL-10 being close to 1; likewise, naïve CD4+ T cells primed by epi-LTA produce lower amounts of IFN-γ and IL-17. In neonatal skin, *S. epidermidis* is critical for the accumulation of CD4+ regulatory T cells, establishing tolerance to skin commensals. Even in low amounts, the inflammatory cytokines induced by *S. epidermidis* can lead to various consequences, such as sepsis, bronchopulmonary dysplasia, white matter injury, necrotizing enterocolitis, and retinopathy of prematurity.

Compared to *S. aureus*, *S. epidermidis* is less pathogenic, and colonization by *S. epidermidis* may even benefit the host local immune system (Summarized in Figure 2). *S. epidermidis* produces small molecules that activate Toll-like receptor (TLR) 2. Exposure of cultured human keratinocytes to a sterile nontoxic small molecule (<10 kDa) isolated from *S. epidermidis*-conditioned culture medium (SECM) increases mRNA expression of antimicrobial peptides (i.e., human beta-defensin 2 [hBD2] and hBD3). It also enhances the capacity of cell lysates to inhibit group A Streptococcus (GAS) and promotes *S. aureus* growth [23]. In mice, SECM administration decreases the susceptibility to GAS infection, confirming that this effect also occurs in vivo. Importantly, this process is TLR2-dependent, as treatment with a TLR2-neutralizing antibody blocks hBD induction, and TLR2-deficient mice do not show induction of mBD4 (mouse ortholog of hBD2). Lipoteichoic acid (LTA), produced by *S. epidermidis* and recognized by TLR2, has proven to be effective in suppressing inflammation. Following skin injury, LTA-induced TLR2 signaling inhibits TLR3-mediated skin inflammation. Specifically, TLR3 signaling is suppressed by induction of TNF receptor–associated factor-1 (TRAF1), a negative regulatory factor that suppresses keratinocyte cytokines (e.g., TNF- α) released via the TLR3 signaling cascade [24]. Lipopeptide 78 (LP78) is another molecule from SECM that has been shown to suppress skin inflammation. LP78 activates TLR2-SRC signaling in normal human epidermal keratinocytes (NHEKs) to induce β-catenin phosphorylation. Phospho-β-catenin translocates into the nucleus to bind to peroxisomal proliferator-activated receptor gamma (PPARγ), disrupting the interaction between p65 and PPARγ. Dissociation of p65 from PPARγ reduces the expression of TLR3-induced inflammatory cytokines in skin wounds of normal and diabetic mice, which correlates with accelerated wound healing [25].

*S. epidermidis* also produces PSMs (PSMγ and PSMδ), which directly induce lipid vesicle leakage and antimicrobial action against skin pathogens such as *S. aureus* [26]. PSMs reduce GAS selectively without affecting *S. epidermidis* growth. Finally, synthetic PSMδ not only binds to and increases the killing capacity of neutrophil extracellular traps, it also binds to host-derived AMPS (i.e., LL-37, CRAMP, hBD2 and hBD3) and enhances their endogenous antimicrobial activity [27].

The Gallo group recently discovered that some strains of *S. epidermidis* also produce 6-*N*-hydroxyaminopurine (6-HAP), a molecule that inhibits DNA polymerase activity. In the context of cancer, 6-HAP inhibits tumor cell proliferation in vitro and B16F10 melanoma growth in vivo [28]. This anti-proliferative activity of 6-HAP is mediated by mitochondrial amidoxime reducing components (mARC1 and mARC2). Importantly, silencing *mARC* with small interfering RNA (siRNA) partially inhibits sensitivity to 6-HAP in non-transformed human keratinocytes. Alternatively, colonization with a 6-HAP-producing *S. epidermidis* strain reduces the incidence of ultraviolet-induced tumors in mice. Thus, further study is needed to examine whether the loss of specific *S. epidermidis* strains increases skin cancer risk in humans, or if increasing *S. epidermidis* skin colonization prevents cancer. Ongoing research is exploring how dysbiosis affects cancer. It has been demonstrated that inflammation induced by dysbiosis promotes carcinogenesis [29,30]. Other observations from the intestinal microbiome suggest that microbes may suppress tumor growth by producing short-chain free fatty acids [29,31]. This study clearly shows that a skin bacterium can protect against neoplasia. *S. epidermidis* or other skin commensals may interact with the tumor environment.

*S. epidermidis* colonization also affects adaptive cutaneous immunity (Figure 2). Indeed, *S. epidermidis* induces IL-17A+/CD8+ T cell homing to the epidermis and enhances barrier immunity [32]. Naik et al. demonstrated that the commensal-driven CD8+ T cell response is antigen specific, and is mediated by a particular subset of dendritic cells (CD103+ dendritic cells). In turn, these IL17A+ CD8+ cells promote skin innate immune responses that protect from cutaneous *Candida albicans* infections. Whether *S. aureus* growth was also suppressed in prior association with *S. epidermidis* was not discussed in the study.

A recent study by the Belkaid group also showed that a specific clade (A20) of *S. epidermidis* induces CD8+ T cell responses. This clade is highly prevalent on human skin, and topical application of the NIHLM087 (from A20 clade) to the skin of non-human primates increases the absolute number of Tc17 cells, but not CD4+ T cells. Hence, the ability of *S. epidermidis* to promote skin CD8+ T cell responses is conserved in non-human primates and likely exists in humans. Furthermore, the researchers specified the characteristics of these CD8+ T cells. Using MHC class I K^b^D^b−/−^ dendritic cells from H2-M3^−/−^ mice that lack MHC class Ia alleles, they demonstrated that *S. epidermidis*-specific CD8+ T cells are confined to MHC class Ib, particularly H2-M3 MHC class Ib. H2-M3-resticted CD8+ T cells are specific to *S. epidermidis*-derived fMet peptide ligands. RNA sequencing also revealed the immunoregulatory and tissue-repair signatures of these CD8+ T cells. In a skin wounding model, *S. epidermidis*-induced CD8+ T cells promoted more rapid epidermal keratinocyte progression, leading to accelerated wound healing.

*S. epidermidis* is also involved in immune tolerance. Tolerance to commensals is crucial for maintaining immune homeostasis at barrier sites. Scharschmidt et al. has created an *S. epidermidis* strain that expresses a model T cell antigen [33]. Using this strain, they discovered that commensal antigens are recognized both locally and systemically across an intact skin barrier. Tolerance to skin commensal bacteria is established in response to neonatal, but not adult, colonization. Tolerance results due to the abrupt accumulation of CD4+ Treg cells between postnatal days 6 and 13. The accumulation of Treg cells is tissue (skin)-specific.

When murine dendritic cells (DCs) were exposed to LTA preparations from *S. epidermidis* (epi-LTA), the ratio of IL-12p70 to IL-10 was close to 1 (IL-10-balanced immune profile), whereas LTA preparations from *S. aureus* (aureus-LTA) induced DCs to produce much higher levels of IL12-p70 (ratio >3). Naïve CD4+ T cells primed by epi-LTA produced significantly lower amounts of IFN-γ and IL-17 compared with those primed by aureus-LTA. The IL-10-balanced immune profile of DCs exposed to epi-LTA was preserved in the presence of the Th2 hallmark cytokine IL-4. Although this should be tested in vivo, we can assume that *S. epidermidis* LTA does not contribute to enhanced cutaneous inflammation in an IL-4-rich environment, but rather regulates the inflammatory response [34]. Taken together, these studies suggest that modulation of *S. epidermidis* can be used to prevent skin diseases.

Despite the favorable results that have been reported for *S. epidermidis*, it remains a major nosocomial pathogen [35]. Risk factors for infection include the presence of indwelling implants, immunosuppression, and increasing antibiotic resistance [36,37]. *S. epidermidis* is also a predominant pathogen of sepsis in preterm infants. Setting aside nosocomial environmental factors, *S. epidermidis* alone is capable of inducing pro-inflammatory cytokine production (e.g., TNF-α, IL-6, and IL-8). Data concerning *S. epidermidis*-induced inflammatory mediators are predominantly based on in vitro experiments and should be studied further in vivo [38].

Multiple studies have reported that the *S. epidermidis* isolated from infections belong to a subset of those found on the skin surface [39]. Using a genome-wide association study (GWAS) approach, 61 genes containing infection-associated genetic elements (k-mers) that correlate with in vitro variations in known pathogenicity traits (i.e., biofilm formation, cell toxicity, interleukin-8 production, and methicillin resistance) were identified. The authors concluded from the data analysis that the disease-causing *S. epidermidis* appears to represent a pathogenic sub-population. These organisms are likely to have acquired genetic elements and related phenotypes that promote infection [40].

Unfortunately, identifying the specific factor or event that drives the transition from harmless commensal to pathogenic factor in skin disease, especially those other than infection, is difficult. Hon et al. examined 100 adolescents with AD and found that *S. epidermidis* was present in severely AD-affected skin lesions, with a positive association to SCORing Atopic Dermatitis (SCORAD) [41]. Alternatively, another recent study by Byrd et al. showed that patients with more mild disease had more *S. epidermidis* detected in flares, and that those with severe disease were predominantly colonized by clonal *S. aureus* strains [42]. Thus, further detailed studies investigating the pro-inflammatory capacity of *S. epidermidis* and its effects on local inflammation and immune priming are needed to determine the role of *S. epidermidis* in inflammatory skin diseases.

## 4. Concluding Remarks

Although *S. aureus* and *S. epidermidis* are not the only *Staphylococcal* species present in the skin, these bacteria are particularly important because they are the most recognized in pathogenic and healthy skin, respectively. Manipulation of these bacteria is likely to significantly affect skin microbiota-host interactions. One way of manipulating the skin microbiota is to introduce specific strains to subjects by topical application. A study has shown that such introduction successfully decreased colonization by *S. aureus*. The result was due to the antimicrobial peptides produced from CoNS [43].

Although these two bacteria share substantial phenotypic properties, their effects on the skin microbiota are contradictory. These differential effects are likely related to different structural adaptations, but those are currently unknown. Additionally unknown are the detailed mechanisms underlying how a specific subset of antigen presenting cells and their epitopes stimulate conventional T cells. Importantly, the skin is a reservoir for tissue-resident memory T cells, which are distinct from recirculating central and effector memory T cells and mucosal-associated invariant T cells. Thus, it is plausible that *S. aureus* and/or *S. epidermidis* affect cutaneous immunity and the homeostatic response through these T cells. Studies evaluating how manipulating these microbes affects various subsets of T cells and long-term local immunity should be performed to discover novel therapeutic strategies. As such, numerous studies are required to understand the complex dialogue between cutaneous microorganisms and host immunity. For now, detailed characterization of the immunogenicity of *S. aureus* and *S. epidermidis* is essential.
